# Data of thematic analysis of farmer׳s use behavior of recycled industrial wastewater

**DOI:** 10.1016/j.dib.2018.09.125

**Published:** 2018-10-04

**Authors:** Loai Aljerf

**Affiliations:** Department of Basic Sciences, Faculty of Dental Medicine, Damascus University, Mazzeh Highway, AlMazzeh, Damascus, Syria

**Keywords:** Thematic analysis, Industrial wastewater, Snowball sampling, Behavioral models, National health services, Green practices

## Abstract

Farmers are concerned in the chemical supply chain (manufacturers, vendors, workers, and consumers) of the agricultural products through their understandings of the safety information (i.e. reading labels such as skull and crossbones symbols, volatile organic compound logo or the fish and tree symbol) and the factors influence misuse of irrigation and disposal behavior. Having recognized a methodological gap, this contribution was intended to investigate qualitatively (textural analysis) the determinants of the use behavior (UB) of farmers irrigating their lands by the recycled industrial wastewater (RIWW) (Aljerf, 2018) [1] using the exploratory investigation based on the single embedded case design. Such combined analytical methods enabled us to achieve both detailed insights into perceptions, behaviors, and an objective understanding of the prevailing opinions that occurred within and between the focus farmers group׳ discussions related around awareness, trust, access and disposal actions within the supply chain. Using the snowball sampling approach, verbal data were collected from 55 Syrian farmers. 5 × 11,000 US gallons (43,900 L) of the RIWW were delivered to each farmer upon request between May and October 2017. After a month of each distribution, the participant farmer was interviewed. To increase the validity of the data, method triangulation was implemented which encompassed participant observation, group debates, and unstructured interviews. The hermeneutic units were analyzed using the pattern-matching method in the Atlas.ti software (version 6.0.15) and the grounded concepts (determinants) were investigated to establish the hypothetical framework at three levels: intrapersonal, interpersonal, and institutional.

**Specifications table**

**Table:** 

Subject area	Chemistry, Ecology
More specific subject area	Chemical Engineering, Analytical Chemistry, and Environmental Chemistry
Type of data	Text file, Tables, Figures, and Supplementary Data
How data was acquired	Field survey, SWOT analysis, and Atlas.ti software (version 6.0.15)
Data format	Raw and analyzed results
Experimental factors	Iterative thematic content analysis based on ethnographic approach was conducted in order to identify deductively and inductively key issues and actions.
	55 farmers were participated in the experiments design and interviewed.
	The potential economic and environmental benefits of the irrigation with RIWW were juxtaposed with stability and safety worries.
	Data extraction, coding and analysis were cross-checked independently using a semi-structured interview schedule based on the theory of planned behavior (TPB) using the Atlas.ti software and the text materials are pieced together to develop the relevant categories.
Experimental features	Analysis levels based on the corroborating Theory/Model i.e. Theory of Planned Behavior (TPB), Innovation Diffusion Model (IDM), and Technology Acceptance Model (TAM).
Data source location	Multiple locations in Syria: Rif Dimashq Governate: Adra Industrial city, 33°35′52″N 36°35′14″E (IWW-treatment, Fig. 1); Maarouneh, 33°37′57″N 36°23′39″E; Alqutayfah, 33°44′02″N 36°37′11″E; Ayn Al-Tina, 33°48′29″N 36°33׳42″E; in addition to Homs (central of Syria): Shinshar, 34°33′57″N 36°44′10″E; Shamsin, 34°34′46″N 36°43′46″E; Maskanah, 34°39′36″N 36°43′36″E; Fairuzah, 34°41′12″N 36°46′57″E; Zaidal, 34°44′45″N 36°46′14″E.
Data accessibility	Data are presented in this article and the Supplementary Data
Related research article	Aljerf [1]

**Value of the data**

• With a fairly care to the local legislation, the collaborated farmers in this Syrian case did not utilize well water treatment system (as for Verotoxigenic Escherichia coli (VTEC) and general waterborne transmission) for irrigation before, so the researcher analyzed the (RIWW-UB) use behavior at three levels i.e. the intrapersonal, institutional, and organizational of wastewater production and inducted the grounded concepts using the exploratory investigation.
• Imitation, habit, and social learning were the main concepts that fitted the social cognitive model (SCM) (Table 1), where understanding societal perceptions was essential to effectively engaging with the consumptive community and informing an improved approach to future pro-environmental engagement and behavior.
• We noticed that environmental impact was neglected or overlooked, possibly due to the fact that programs as fertilization and clean irrigation are generally not funded and pollution prevention programs require a shift in thinking. That is why farmer׳s capacity engagement in a regular risk management was reduced by limited perceptions of risk susceptibility and severity, impeding cues to action and barrier concerns. So, the solution could be prescribed by enhancing factors granted as financial support, training programs (i.e. sustainable environmental management practices, change behavior practices), rewards systems for irrigation with clean-recycled wastewater and improvement of standards.
• The use of RIWW had qualitative-, legal- (i.e. eco-tax payment) and financial aspects, in addition to stakeholders as farmer׳s involvement with effective cooperation as a joint-force.
• Farmers had expectations for high-quality water treatment conducted in a manner that increases their products׳ consumer confidence.

## 1 Data

There is no paper discussed any aspects of RIWW management in the literature including its use intensity, use behavior (UB), and decisions about its reusage. In addition, there is no paper has provided the quantitative and cross-sectional data attained by even simple methods and tools, i.e. survey and structured questionnaires. The primary data were collected which helped to make a SWOT (Strength, Weakness, Opportunity, and Threat) analysis of the developed method (Table 2).

**Fig. 1:**
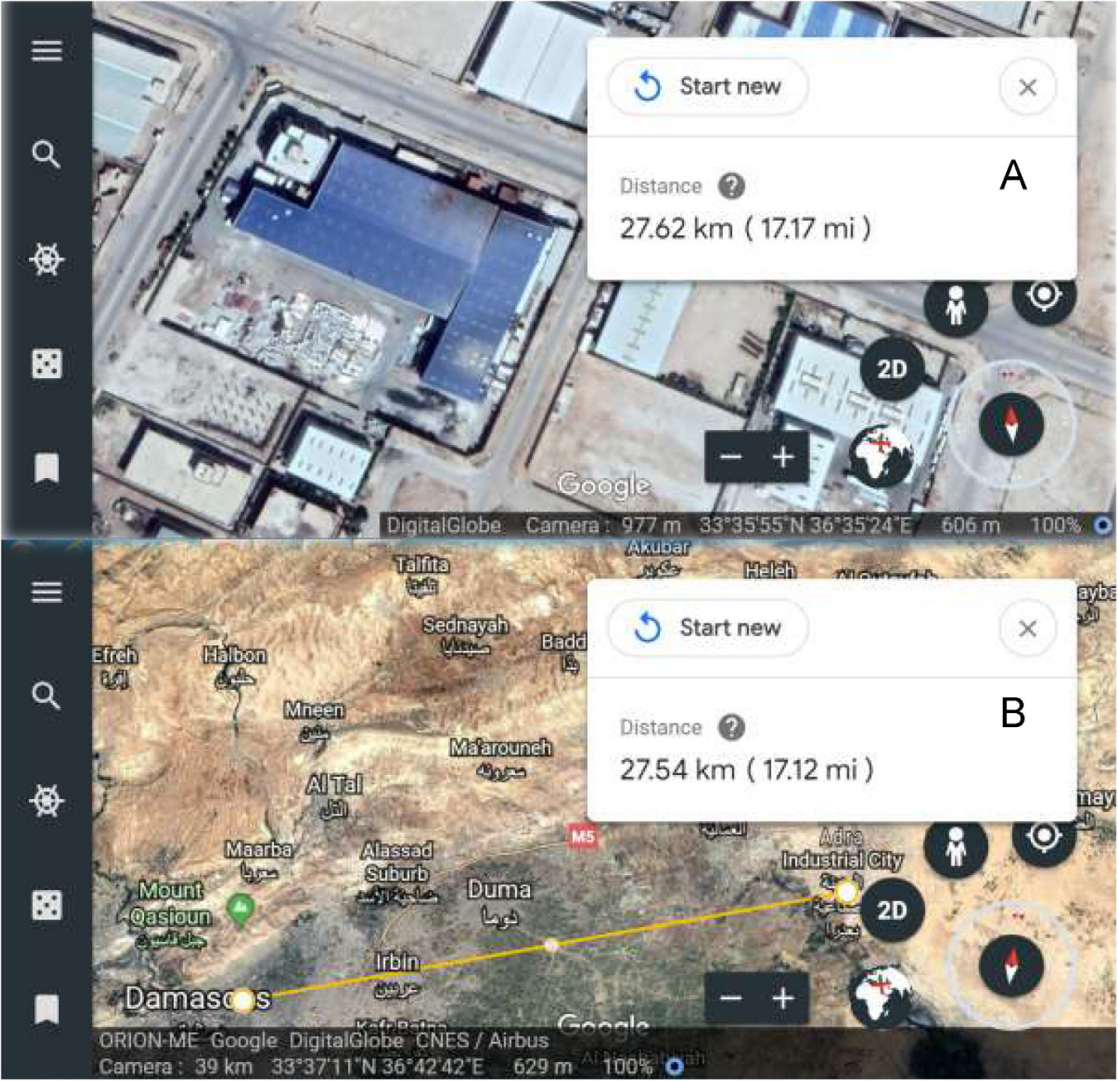
Using KML (GoogleMaps), (A) Industrial case study (dyestuff), (B) geographical location of the industrial wastewater (IWW) collection involving its treatment for next reuse in irrigation sector.

**Table 1:** SWOT analysis and recommendations for the use of RIWW to promote the development and use of new-innovative environmentally beneficial technology.

Method strengths (Specific-inherent characteristics)	Weakness	Opportunity and applicability	Threats	Recommendation
Simple, easy, and of low administrative costs	When the conditions are not completely practiced	When differences in the marginal costs of industrial pollution abatement are small and economically feasible solutions to environmental problems are available	When there is a lack of environmental consciousness	Communication (e.g. ecolabels) helps to focus the attention of industrial firms and consumers on environmental problems and the applied solution to these problems
*Technology-based environmental standards.*		*When there is a consensus about an appropriate compliance technology*	*When there are information failures*	
Effective in most aggregation cases		When there is uncertainty about best solution.	Controversies about problems and solutions	
*Effective in focusing industry’s minds on environmental problem*		*When there is uncertainty about industry response*		*Network management creates a platform for learning and interaction, to stimulate alignment coordinate, interdependent activities solutions may be tailored to specific needs*
Environmental benefits and technological opportunities are available and are developed at low enough costs		Uncertainty about whether industry will meet agreements		
*Recycling and energy saving*		*When environmental performance is expensive*		
Good technological diffusion and incremental innovation		When markets for environmental technology do not yet exist and when there is uncertainty about future policies		Societal debates about environmental issues associated with these industries
*Tradeable permits*		*In case of large social benefits and insufficient private benefits*		
Political attractiveness		In conflict with polluter-pays principle		*Sustainability foresight projects*[2]*broaden our developed processes of ecological assessment and enhance strategic orientation*
		*Demanding of eco-tax efficiency*		
		Danger of windfall gains politically expedient		
		*In case of heterogeneous pollution in the RIWW which respond to price signals*		
		When there are important knowledge spillovers		
		*When industry suffers a competitive disadvantage due to less strict regulations in other countries*

**Table 2:** The compositional structure of the consequences of RIWW reuse category.

**Consequences of RIWW reuse**
Farmers׳ attitudes towards RIWW reuse involved an evaluation of the benefits and the risks associated with the distribution of returned pollutants to consumers:
***Potential advantages of RIWW reuse***
A. Economic impact on the national health services administration followed by the ministry of health (NHSA-MH).
■ Direct monetary savings for the NHSA-MH. ■ Reduction in agricultural expenditure. ■ Cost-benefit of reusing cheaper water.
B. Environmental effects
■ Reduction in negative environmental effects of water disposed inappropriately. ■ Reduction in the carbon footprint.
***Potential disadvantages of RIWW reuse***
A. Poor quality water
■ Cleanliness of the storage environment (tanks and reservoirs).
B. Harmful effects
■ Deliberate or malicious tampering with returned pollutants. ■ RIWW can be as a source of infection if contaminated and does treated efficiently as using Aljerf method [1].
C. Incorrect usage
■ Errors introduced by farmers. ■ Errors introduced by transportation. ■ Risk posed by accepting counterfeit source of RIWW.

## 2 Experimental design, materials, and methods

### 2.1 SWOT analysis

See Table 1. 

### 2.2 Method triangulation

**Fig. 2:**
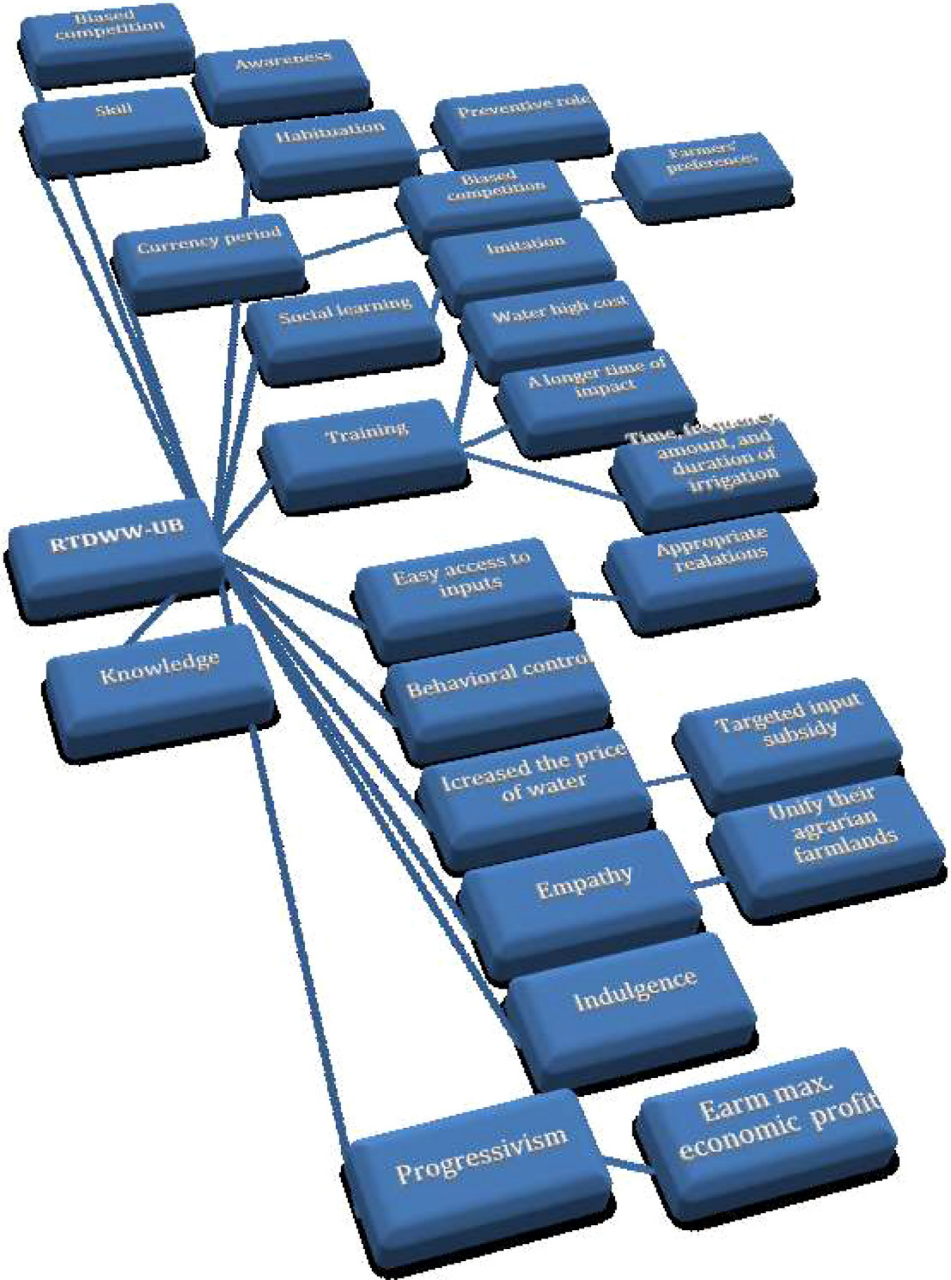
The sequence of 55 interviews with respondents on the topic of the RIWW-UB.

The interviewees were allowed to unburden whatever perceived about the RIWW use and the unstructured interviews were registered. In this regard, new questions were designed and updated continually. To understand the RIWW-UB, the method triangulation have been implemented, i.e. the multiple sources of evidence that include (i) unstructured interviews, (ii) group debates, and (iii) participant observation. Moreover, the hermeneutic units were analyzed using the pattern matching approach in the Atlas.ti software (version 6.0.15). The text materials were pieced together to expand the relevant categories. Then, more detailed analysis of the characteristics and relations among the categories using the conceptual focusing were developed. Fig. 2 shows the sequence of the grounded concepts and determinants of the RIWW-UB.

### 2.3 Method boundary for categorizing RIWW appeal

The classifications of the RIWW appeal are described in details in Table 2, Table 3, Table 4.

**Table 3:** Compositional structure of ‘Exemplar and anti-exemplar individuals and groups’ category.

**Exemplar and anti-exemplar individuals and groups**
The groups of individuals or people whom the participants thought would or would not engage with and approve of RIWW reuse
***Individuals or groups of people who approve of RIWW reuse***
A. The Green movement
• Farmers׳ families, partners, relatives, and friends who ‘think green’. • Environmentalists. • The Green Party, the political organization.
B. The elderly
● Those with a dislike of waste and an affinity for frugality.
***Individuals or groups of people who disapprove of RIWW reuse***
A. Water companies
• Employees. • Beneficiaries.
B. Taxpayers
● Taxpayers with a sense of entitlement (i.e. feudal lord).
C. Vulnerable individuals (those making a decision for them)
• Babies. • Children.
D. The elderly
• Cautious individuals worried about safety. • Terminally ill-patients.

**Table 4:** The compositional structure of ‘expectations about returned RIWW category.

**Expectations about returned RIWW for irrigation purposes**
Factors that may facilitate or impede the workability of RIWW reuse by farmers
***Physical characteristics of returned RIWWW***
A. Reservoirs
➢ Materials used potentially and appropriately for filling water reused.
B. Whether the reservoirs had been opened or not
➢ Only unopened reservoirs to be used.
C. Remaining shelf-life of reused water
➢ Water should have less than a month of shelf life if to be reused according to our experiences in this type of treated water.
D. Physical reused RIWW characteristics
➢ Suspended particles in the treated water including its turbidity not to be reused.
***The quality assurance of returned RIWW***
A. Storage conditions
➢ Cleanliness of the storage environment and risk of spread of infection.
B. Damaged water
➢ Accidental toxication to the reused RIWW.
C. Counterfeit RIWW
➢ RIWW bought from untrusted sources including online sources not to be reused.
***The logistics of RIWW reuse***
A. Collection and redistribution of RIWW ‘on-site’ within a water system setting
➢ Efficiency of resusage system of RIWW. ➢ Space for collection, processing, and storage of the RIWW. ➢ Hydrologists׳ time availability to conduct quality assurance of the RIWW.
B. Collection and redistribution of RIWW ‘off-site’
➢ Collection spots within the IWW-treatment plant unit (Fig. 1). ➢ Water inspection centers responsible for checking water for reusability. ➢ Water companies to be involved in funding and supporting reuse processes.
C. Incentives for taking part in RIWW reuse
➢ Points reward system to encourage the return of RIWW after completeness of the industrialization process. ➢ Discount on water tax in industry to encourage the reuse of RIWW.

### 2.4 Farmers’ appeal of the RIWW-UB

#### 2.4.1 Behavioral models

The behavioral models were reviewed which have a potential to explain the RIWW-UB.

##### 2.4.1.1 Theory of Planned Behavior (TPB)

**Fig. 3:**
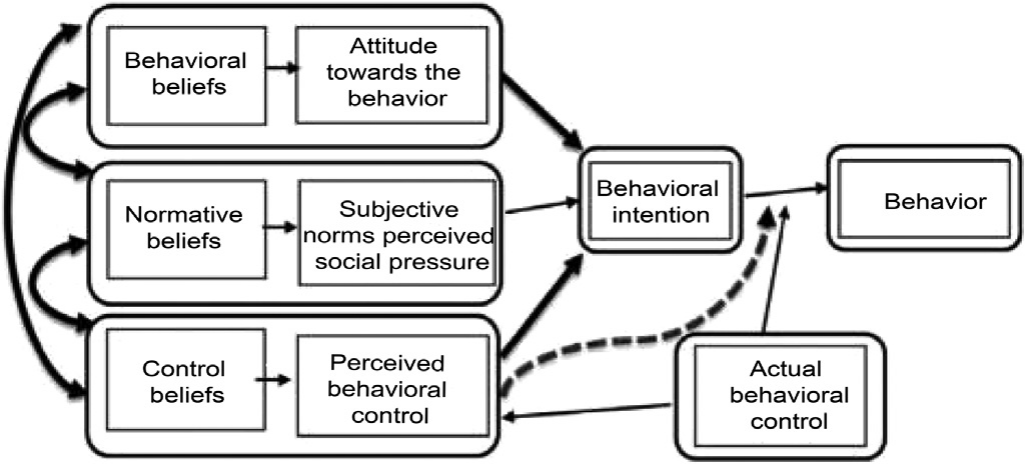
Schematic representation of the theory of planned behavior (TPB) showing the relationship between the determinants of behavior.

Constructs in the TPB (Fig. 3) were not adequate to investigate the pro-environmental behaviors because the adoption of an environmental behavior was associated with the values and ethical frameworks. We had extended and added up new concepts to the TPB, e.g., moral norm, environmental values, environmental consciousness, social identity, environmental concern and knowledge, moral obligation, and habits. These concepts (Table 5) were affected the intentions and the behaviors of the farmers. The environmental ethics, beliefs, and consciousness impacted the adoption of green practices in agriculture.

**Table 5:** Comparative conceptual analysis.

##### 2.4.1.2 Innovation Diffusion Model (IDM)

The method of diffusion process was designed to include four key components: (A) innovation, (B) communication channel, (C) time, and (D) social system. The perceived innovation characteristics that influenced adoption were the advantage, testability, compatibility, complexity, and observability. Furthermore, the innovation-decision process consisted of five points: knowledge, persuasion, decision, implementation, and confirmation. The variables that influenced farmer׳s knowledge were personality characteristics, e.g., the general attitude towards change, social characteristics, e.g., cosmopolitanism, and perceived need for innovation. The social system also included social norms, tolerance of deviance, and communication integration (Table 5).

##### 2.4.1.3 Technology Acceptance Model (TAM)

This proposed model was connected to the perceived ease of use (PEU) and perceived usefulness (PU) which affected the intention to use (ITU) directly (Table 5).

##### 2.4.1.4 Social Cognitive Model (SCM)

According to the SCM (Table 5), the behavior is affected by the reciprocal interaction of the environmental (i.e. farmer׳s physical environment, reinforcement, observational learning), personal (i.e. outcome expectations, outcome expectancies, self-efficacy expectations), and behavioral factors (i.e. self-observation, self-judgment, and self-reaction).

#### 2.4.2 Conceptualizing RIWW use and the use behavior

##### 2.4.2.1 Analysis levels of behavior

Attitudes, values, beliefs, and motives were perceived as the operating factors at the *individual* level of the farmers.

###### 2.4.2.1.1 Personal characteristics

These represented the intrapersonal processes (Table 5), e.g., decision-making, personality differences, and acquired competencies. Between these characteristics are:

A. *Literacy, knowledge, and awareness*
B. *Progression-centered preferences*
C. *Avarice.*

###### 2.4.2.1.2 Organization of RIWW reusage

I. Sympathy for farmers
  Most farmers just paid attention to their economic profitability and did not regard the environmental protection and health issues. Farmers were less empathy for others, as they frequently produced polluted agricultural products due to their misunderstanding of the human and physical environment (Supplementary Data). The direct outcomes of the UB hardly were observable for farmers since they had not a complete vision to imagine the consequence of the UB.
II. *Trust* (*see* Table 4)
  Associated with the UB, high-quality RIWW [1] use started by providing farmers the accurate information that made them trusting the information provider (we) as one of the stakeholders in the supply chain.
III. *Farmers׳ associations (FAs)*
  The Farmers׳ associations (FAs) were found suitable to interact with the other organizations and campaigns that could affect farmer׳s UB.
IV. *Farm characteristics*

Farm characteristics as arable soil quality, farm type, machinery, off-farm labor, and farm size were affected the RIWW overuse.

###### 2.4.2.1.3 Institutional

(i) *Agricultural input subsidies*
(ii) *Checking-up RIWW characteristics*
(iii) *Training*
(iv) *Perceived uncertainty*

Under uncertain conditions, farmers made ex-ante decisions that impacted on their incomes on the one hand and on the risks on the other hand. Uncertainty was related to the unpredictability of the farm situations. Therefore, a remarkable part of the uncertainty relevant to the RIWW use was the fact that farmers could not see the overt consequences of this kind of water use. For this reason, they perceived the use of this water as an important and permanent way.
