# Supplementary material for: Data of thematic analysis of farmer׳s use behavior of recycled industrial wastewater
Source: Data Brief. 2018 Oct 4;21:240–50. doi: 10.1016/j.dib.2018.09.125 (PMC6197390; doi:10.1016/j.dib.2018.09.125)
Supplement: Supplementary file 1 — Supplementary material. [file mmc1.docx]

**Conflict of interest form**

**Conflicts of interest**

The author declares no conflicts of interest.

**Funding**

No external funds. So, no funders have any role in the study design, in the collection, analysis and interpretation of data, in writing of the report, and in the decision to submit the article for publication.

**Contributors**

The author puts the design of this study and directed its implementation, including data analysis, and writing the paper.

**Submission declaration**

The author declares that the work described has not been published previously, that it is not under consideration for publication elsewhere, and it will not be published elsewhere including electronically in the same form in English or in any other language without the written consent of the copyright-holder. In addition, the corresponding author has consulted the Guide for Authors in preparing this submitted manuscript and confirms that this work has been prepared in compliance with the Ethics in Publishing Policy as also described in the Guide for Authors.
