# Supplementary material for: Data of thematic analysis of farmer׳s use behavior of recycled industrial wastewater
Source: Data Brief. 2018 Oct 4;21:240–50. doi: 10.1016/j.dib.2018.09.125 (PMC6197390; doi:10.1016/j.dib.2018.09.125)
Supplement: Supplementary file 2 — Supplementary material. [file mmc2.pdf]

Supplementary Information

Data article

Data of thematic analysis of farmer's use behavior of Recycled Industrial Wastewater

Loai Aljerf*

Department of Basic Sciences, Faculty of Dental Medicine, Damascus University, Mazzeh Highway, AlMazzeh, Damascus, Syria

Appendix A

Participant's Code Number:

Household Code:

Date and time visited:

Background Information

Gender–Female/Male

Questions

– How many farmers are working in the farm including

* Corresponding author. E-mail addresses: envirochrom@hotmail.com; loai.aljerf@aol.com

you?

– If you shop as a household, individual or both how do you decide what food to buy?
– When shopping as a household, individual or both, do you buy certain food on special offer knowing that you might never eat it? Yes/No
– When eating as household, individual or both, how do you decide what you are going to eat?
– Do you smell the vegetables and fruits before buying? Yes/No
– Do you smell the vegetables and fruits in your field before you sell them? Yes/No
– Do you read labels? Yes/No
– Do you recognize that some foods are polluted? Yes/No
– Have you read the topic of 'polluted agricultural products'? Yes/No
– Do you recognize that some pollutants come from the irrigation with impure water? Yes/No
– What does the best before date of irrigation on a specific agricultural waste indicate?
– What does the irrigation by date on a specific agricultural waste indicate?
– What do you do when an agricultural product goes past the best before date on the label?
– What do you do if an agricultural product goes past

the use before date on the label?

– Do you believe you are wasting money when throwing agricultural products away? Yes/No

– Are you aware of the monetary value of wasted agricultural products? Yes/No

– Could you calculate the monetary value of the wasted agricultural products you throw away? Yes/No, if so, on a weekly basis, what is the monetary value of these products you throw away?

– Are you aware of the monetary value of irrigating with impure water? Yes/No

– Are you aware of the monetary value of polluted agricultural products that you sell every year? Yes/No

– Are you aware of the monetary value of stack of polluted agricultural products daily purchased from the market by you or any other consumers? Yes/No

– Have you ever thought that the cattle in your farm could be largely polluted? Yes/No

– Have you ever thought that your crop could be largely polluted? Yes/No

– Is saving money a motivation that encourages you to waste fewer products? Yes/No

– Are you aware of the environmental impacts of agricultural waste products? Yes/No

– Are you aware of the environmental impacts of

polluted crops? Yes/No

– What do you consider to be the most important aspects?

– Are you aware of the social impacts of agricultural waste? Yes/No

– Are you aware of the social impacts of polluted crops? Yes/No

– Are you aware of the social impacts of the recycled industrial wastewater (RIWW) if reused for irrigation? Yes/No

– What do you consider to be the most important aspects?

- As a farmer and consumer of other products at the same time, what is the primary factor that would encourage you to waste less of the food that you or one of your family could be a victim to these selling products?
- What other factors would encourage you to waste less food?
- Do you support the idea of decentralization of water sector? Why?
- When you hear the phrase "polluted agricultural products", what comes to mind?
- In your view, are polluted agricultural products by irrigation with wastewater or any other source of impure water an issue that would affect the community at large?

- What do you think can be done to manage this issue?
- Can you think of things that you can do as a farmer that can help in reducing the pollution of agricultural products by misuse of untrusted source of water in irrigation?
- Have you read or heard about any of the following terms: 'water footprint (WFP)', 'water security (WS)', 'environmental performance (EP)', 'sustainable development (SD)', 'rural sustainability (RS)', 'sustainable consumption and production (SCP)', and 'cleaner production (CP)' of irrigation water? Yes/No. If yes, give examples about what you read and/or heard?*
- Do you think that the current project can draw farmer's lifecycle thinking (LCT)** towards sustainable countryside?
- As a result of this interview what steps, if any, are you prepared to take to reduce polluted agricultural products?
- Are you satisfied for irrigation with RIWW? Yes/No and Why?

---

* Farmers who showed a good awareness about any of these issues were asked to estimate if the current topic satisfies and/or serves the principle(s) of the recognized term(s).

** This expression was explained to farmers.

1. Reuse schemes of water monitoring

Table S1 is designed according to the experience of the researcher in this domain.

Table S1

Frequencies for any monitoring scheme used for assessing of a potential source of impure water for irrigation.

Parameter	Monitoring on-line	Monitoring for performance	Monitoring for health risk/safety
pH	4	5	
Turbidity	5	7	4
TSS			2
Conductivity	1	4	
Total *Coli*		5	4
Fecal *Coli*		1	3
Nematodes			
Streptococcus			
Micro pollutants			
Total residual chlorine	5	4	4
Energy	2		
Chemical use	1		
Flow	4		
Temperature	2		

Odor		1	1
Oil and grease		1	1
Nitrogen	2	4	2
Ammonia		2	
Phosphorous	2	4	2
BOD		4	2
MLSS		3	
DO		2	
NFR		1	
TDS		3	

2. Farmer awareness of the social impact of RIWW

Table S2

Some preliminary indicative comments considered as 'influencing factors' which are registered by respondents.

'I cannot visually see the monetary value of agricultural products' waste'.
'Today's farmers do not mind wasting food by irrigation with polluted water, as it is so readily available and cheap, as I am the same. But if food was to become more expensive, then everyone would be encouraged to waste less food, as they will not be able to afford to waste food'.
'I fail to comprehend the monetary value of wasting food adds up'.
'I know what the 'use by' stands for but not what the 'best before' stands for'.
'If food becomes more expensive, I would definitely start wasting less food'.
'Food safety concerns regarding meat but not vegetables'.

‘Why cannot manufactures get rid of the ‘best before’ date and only have a reasonable ‘use by’ date or if a product never goes off like pasta or rice, then have no date at all. It is almost like food manufacturers (Not Farmers) are trying to get consumers to waste huge amounts of food, as we are not all educated in food and waste of land’.

‘Vegetables wasted, as perceived cheap, but not meat, as seen as the most expensive part of a meal’.

‘If there was not the safety risk related to food product, I would definitely waste less products, but I am far too scared to risk it, so it is just easier to throw polluted agricultural products away (involving waste), rather than having food poisoning’.

‘I have a desire for a long-term and apolitical approach to managing water for irrigation’.

‘Water quality is important when considering end uses’.

‘Water is so precious; bad agriculture wastes water’.

‘We are in need of a bank account for high-quality water for irrigation’.

‘If I knew the exact total amount of the monetary value of the food I waste I waste was, then probably yes’.

‘Wasting food does not waste money’.

‘I think what we do need to evaluate, and to get a better grip of, is some of our unknown territory in public health’.

‘I do not think I have ever had any formal training on evaluation. But, what I have done is learnt on the role and I have been…I have looked for examples of best practice and stuff elsewhere’.

‘It has to be related to outcomes, and it has to have an economic dimension to it. And then you know…I think that is absolutely critical because that is all I get asked for nowadays is economic stuff’.

‘Most of the time, I will be honest with you, it is searching online, it is looking at the

relevant websites… and taking elements of various evaluations and designing them myself'.

'I think it is one thing producing a resource, but it is the challenge is getting it useable again for the farmer that you are aiming it at, and you cannot…I mean there is no hope of getting it used if it is not going hit the right person'.

'I think if there is national guidance on what is expected in an evaluation of water, that might be useful, then everybody is working to the same document. Because, I think there are lots of local things floating around that only a few people have access to, so having a national one there everybody in public health can access'.

'Even if you commission it, you still have to know, what you are commissioning…you know, you still have to know what is expected from a qualitative survey? What is expected from a quantitative survey and so on and so forth?'

'… So, we need to look after what we have, and not just hope that the next breakthrough is just around the corner. I certainly hope that it is. But … where does it stop? We might find a new source of cheap water, but of high-quality, well that one becomes appealed… so, I think it is a continual thing'.

'I used to lose 9-23% of water in pipe networks during irrigation'.

'Usually we consume 60-70% of water for irrigation in this region, in addition to, 14-26% of chemicals, and 4-26% incombined wastewater'.

'I have the impression that large volumes of IWW are wasted without recycling which need the application of a developed method as yours'.

'I need 800 L of RIWW per kg of corn'.

'After irrigating my land with RIWW, I became aware of the necessity to promote efficient waste- and greywater treatment and recycling alternatives as of industrial source'.

‘Desalination units average costs \$0.69-1.18/m^3, however, the transport fees of RIWW is \$0.027/m^3 which sounds competitive’.<br>‘I think to convey the RIWW in a cleaner modernized method; we need to use of intelligent transport systems (ITS)’.
